# Quantifying the advantage of domain-specific pre-training on named entity recognition tasks in materials science

**DOI:** 10.1016/j.patter.2022.100488

**Published:** 2022-04-08

**Authors:** Amalie Trewartha, Nicholas Walker, Haoyan Huo, Sanghoon Lee, Kevin Cruse, John Dagdelen, Alexander Dunn, Kristin A. Persson, Gerbrand Ceder, Anubhav Jain

**Affiliations:** 1 Energy Technologies Area, Lawrence Berkeley National Laboratory, 1 Cyclotron Road, Berkeley, CA 94720, USA; 2 Materials Sciences Division, Lawrence Berkeley National Laboratory, 1 Cyclotron Road, Berkeley, CA 94720, USA; 3 Molecular Foundry, Lawrence Berkeley National Laboratory, 1 Cyclotron Road, Berkeley, CA 94720, USA; 4 Department of Materials Science and Engineering, University of California, Berkeley, 210 Hearst Memorial Mining Building, Berkeley, CA 94720, USA

**Keywords:** NLP, NER, BERT, transformer, language model, pre-train, materials science, solid-state, doping, gold nanoparticles *PACS:* 07.00.00, 81.00.00

## Abstract

A bottleneck in efficiently connecting new materials discoveries to established literature has arisen due to an increase in publications. This problem may be addressed by using named entity recognition (NER) to extract structured summary-level data from unstructured materials science text. We compare the performance of four NER models on three materials science datasets. The four models include a bidirectional long short-term memory (BiLSTM) and three transformer models (BERT, SciBERT, and MatBERT) with increasing degrees of domain-specific materials science pre-training. MatBERT improves over the other two BERT_BASE_-based models by 1%∼12%, implying that domain-specific pre-training provides measurable advantages. Despite relative architectural simplicity, the BiLSTM model consistently outperforms BERT, perhaps due to its domain-specific pre-trained word embeddings. Furthermore, MatBERT and SciBERT models outperform the original BERT model to a greater extent in the small data limit. MatBERT’s higher-quality predictions should accelerate the extraction of structured data from materials science literature.

## Highlights

• Efficient extraction of information from materials science literature is needed
• Domain-specific materials science pre-training improves results
• Even simpler domain-specific models can outperform more complex general models

## The bigger picture

A bottleneck in efficiently connecting new materials discoveries to established literature has arisen due to a massive increase in publications. Four different language models are trained to automatically collect important information from materials science articles. We compare a simple model (BiLSTM) with materials science knowledge to three variants of a more complex model: one with general knowledge (BERT), one with general scientific knowledge (SciBERT), and one with materials science knowledge (MatBERT). We find that MatBERT performs the best overall. This implies that language models with greater extents of materials science knowledge will perform better on materials science-related tasks. The simpler model even consistently outperforms BERT. Furthermore, the performance gaps grow when the models are given fewer examples of information extraction to learn from. MatBERT’s higher-quality results should accelerate the collection of information from materials science literature.

## Teaser

Efficient automated extraction of information from materials science literature is needed due to an increasingly unwieldy number of publications. For a selection of materials science NER tasks, Trewartha et al. find that language models pre-trained on materials science literature provide measurable advantages over language models pre-trained on general literature or even scientific literature from multiple fields. This provides an opportunity to produce higher-quality results that require less training data in order to address this problem.

## Introduction

Recently, the number of publications in the field of materials science has grown exponentially.^1^ As a result, it has become increasingly difficult for researchers to follow research progress as it emerges, even within relatively restricted sub-domains. The size of the materials science literature means that even relatively simple questions, such as which material candidates have previously been studied for a particular application, can be difficult or impossible to comprehensively answer. This has created a need for new, more efficient ways to engage with the literature and extract the relevant information therein.

Natural language processing (NLP), the analysis of unstructured text using computers, provides a natural candidate for such an alternative approach. NLP has successfully been applied to a number of materials science applications and is the topic of several recent investigations in materials informatics.^2^, ^3^, ^4^, ^5^ Additionally, work has been done to develop meta-learning strategies for NER.^6^, ^7^, ^8^ Recently, the advent of transformer ML architectures such as BERT^9^ have revolutionized NLP; leading benchmarks such as GLUE^10^ are now dominated by models utilizing attention-based encoder-decoder architectures called transformers^11^ and perform comparably to humans on some tasks. Transformer models have ushered in a new NLP paradigm where large and general NLP models are “pre-trained” on semi-supervised tasks before being fine-tuned for downstream tasks.^9^^,^^12^, ^13^, ^14^, ^15^, ^16^, ^17^ The pre-training approach allows for task-specific models to be trained using relatively few hand-annotated examples; this is a useful feature for practical applications of NLP bottlenecked by annotation such as scientific tasks that contain technical text and esoteric vocabulary.

Although a single pre-trained model may address multiple NLP tasks (e.g., question answering, named entity recognition, next sentence prediction), the success of models with domain-specific pre-training such as BioBERT,^18^ CaseHOLD,^19^ and FinBERT^20^ begs the question: can transformer models be further improved with even *more* domain-specific pre-training? We hypothesize that the measurable advantages previously shown with domain-specific pre-training—for example, of SciBERT over BERT^21^—can again be extended to models specific to narrower scientific disciplines such as materials science. Improved domain-specific model performance implies improved ability for automated knowledge extraction from even the most complex and vexing (from the perspective of NLP models) scientific domains. Exploring this problem in-depth presents an opportunity for the collation and synthesis of massive numbers of highly complex scientific publications into otherwise inaccessible structured databases and models for knowledge generation.

In this work, we apply transformer models to the task of named entity recognition (NER)^22^ to extract and label important scientific entities relevant to materials chemistry from unstructured text. A well-trained NER model will be capable of automatically mapping the unstructured text of materials science publications to a queryable database of key terms. Historically, NER has been used to extract information such as names and locations from various articles, though recently it has been employed in the chemical, medical, and materials sciences as well.^1^, ^2^, ^3^, ^4^^,^^23^, ^24^, ^25^, ^26^, ^27^, ^28^, ^29^, ^30^, ^31^, ^32^, ^33^, ^34^, ^35^, ^36^, ^37^, ^38^, ^39^ For material science, this may include terms that refer to materials and their geometries, properties, syntheses, methods of characterization, and downstream applications. Strongly related work in text mining and language modeling has also been employed in the same fields.^5^^,^^40^, ^41^, ^42^, ^43^, ^44^, ^45^, ^46^, ^47^, ^48^, ^49^, ^50^, ^51^, ^52^, ^53^, ^54^, ^55^, ^56^, ^57^, ^58^, ^59^, ^60^ BERT has additionally found use in biology, medicine, and materials science.^18^^,^^61^^,^^62^

Specific to the field of materials science, there have been significant efforts to apply NER to the extraction of materials synthesis recipes, including using BERT.^2^^,^^28^^,^^29^^,^^57^^,^^62^ In the past, these have employed a combination of the aforementioned work in the chemical sciences to extract inorganic material entities with syntax trees and lookup tables to extract properties and processing conditions. The recently developed transformer-based models have been shown to offer significant performance improvements on NLP tasks.^9^ This provides an excellent opportunity to evaluate the performance of these new models on NER tasks specific to materials science.

In this work, we apply four different NER models to three different materials science datasets and analyze their performance. The simplest model considered is a bidirectional long short-term memory (BiLSTM) recurrent neural network. The other three models, variants of the popular transformer-based BERT_BASE_ neural network structure,^9^ have identical model structures but use pre-training corpora of varying domain specificity. The considered datasets consist of one general-purpose materials science dataset (referred to as the solid-state dataset) and two topic-specific datasets that respectively focus on doping and gold nanoparticle synthesis. We use the results of NER on these materials science datasets to relate the domain specificity of the pre-training corpus to measurable performance differences in extracting named entities.

### Datasets

Here we consider three different NER datasets, chosen to represent a diversity of text sources and problems relevant to materials science; a set of solid-state materials science abstracts with entities of broad interest,^28^ a set of abstracts with inorganic doping information, and a set of methods/results sections relevant to gold nanoparticle synthesis. Each of these is described in detail below. The solid-state dataset is publicly available,^63^ though only the DOIs and annotated entities are available for the other two.^64^

#### Solid-state dataset

The solid-state dataset discussed in this work consists of 800 annotated abstracts from solid-state materials publications collected using Elsevier’s Scopus/ScienceDirect^65^ and Springer-Nature^66^ APIs as well as web scraping for journals published by the Royal Society of Chemistry^67^ and the Electrochemical Society.^68^ Abstracts are considered relevant if they mention at least one inorganic material and at least one synthesis or characterization method for inorganic materials. The entity labels are chosen to represent a broad domain of materials science knowledge with eight different labeled entity types: inorganic materials (MAT), symmetry/phase labels (SPL), sample descriptors (DSC), material properties (PRO), material applications (APL), synthesis methods (SMT), and characterization methods (CMT). Details of the collection and pre-processing of these abstracts and detailed definitions of the labels are available in Weston et al.^28^

**Figure 1:**
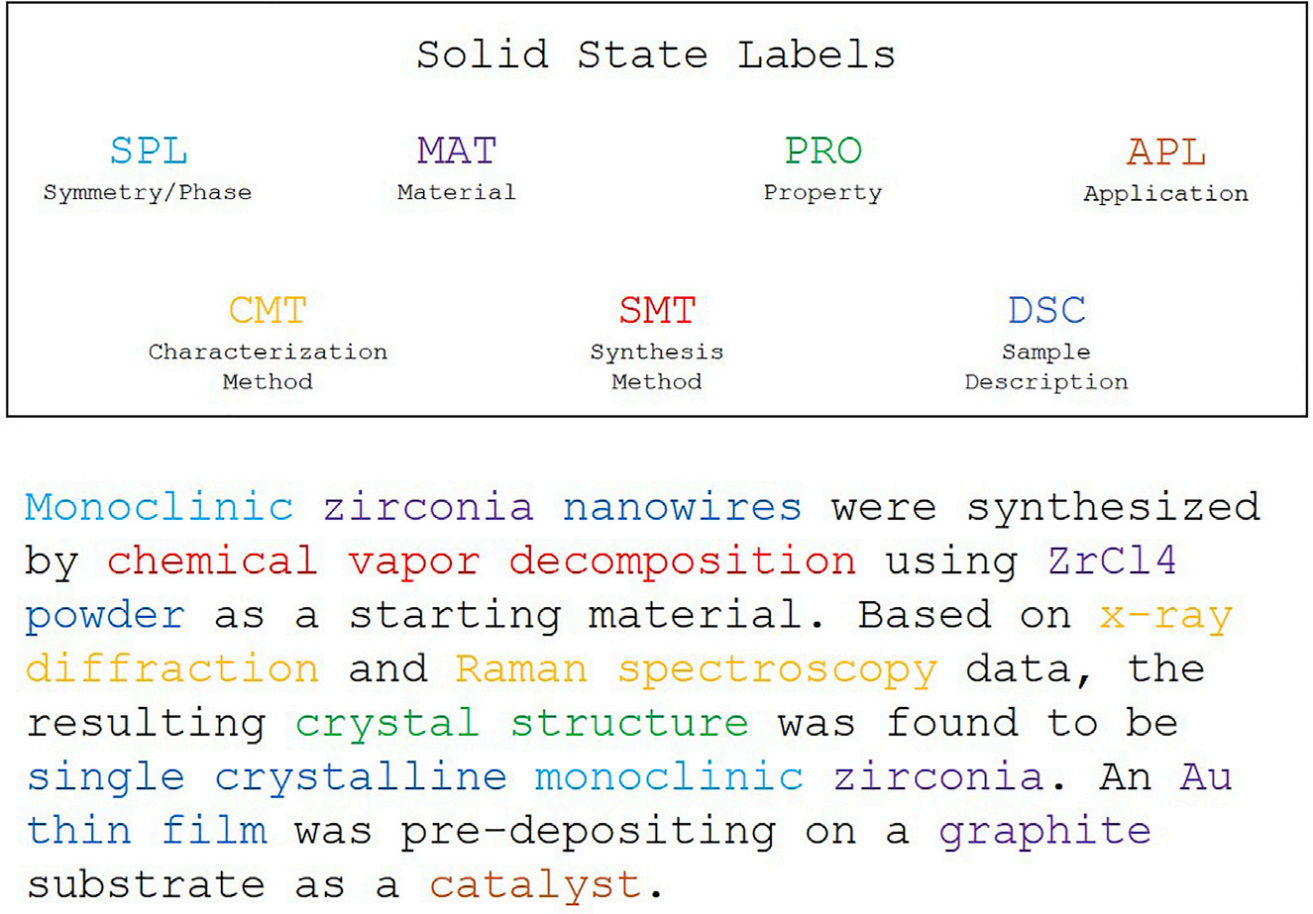
Solid-state annotation example An example of the solid-state annotation scheme condensed from an example abstract in the solid-state dataset.

A condensed example is shown in Figure 1.^69^ This dataset is intended to provide a “catch-all” of relevant information without focusing on any specific facet of solid-state materials. Due to the broad definitions of the entities, the solid-state dataset generally contains more entities per paragraph than the other datasets. Additionally, an inter-annotator agreement of 87.4% was evaluated utilizing 25 annotations from a second annotator.^28^

#### Doping dataset

The properties of doped materials used for applications requiring semiconductors are determined by critical pieces of information such as the base material (BASEMAT), the doping agent (DOPANT), and quantities associated with the doped material such as the doping density or the charge carrier density (DOPMODQ). The intention of this dataset is to capture the information relevant to the doping of a material and any other relevant quantitative measurements. Abstracts that specifically contain information about doping, i.e., those containing regular expressions matching “dop∗” (such as “dopant,” “doped,” and “co-doping”) or “n-type” or “p-type,” were queried from the Matscholar database of materials science abstracts.^70^ A set of 500 abstracts was randomly sampled from the queried set, from which 455 abstracts were identified by human annotators as relevant to inorganic materials science and were annotated by three annotators.

**Figure 2:**
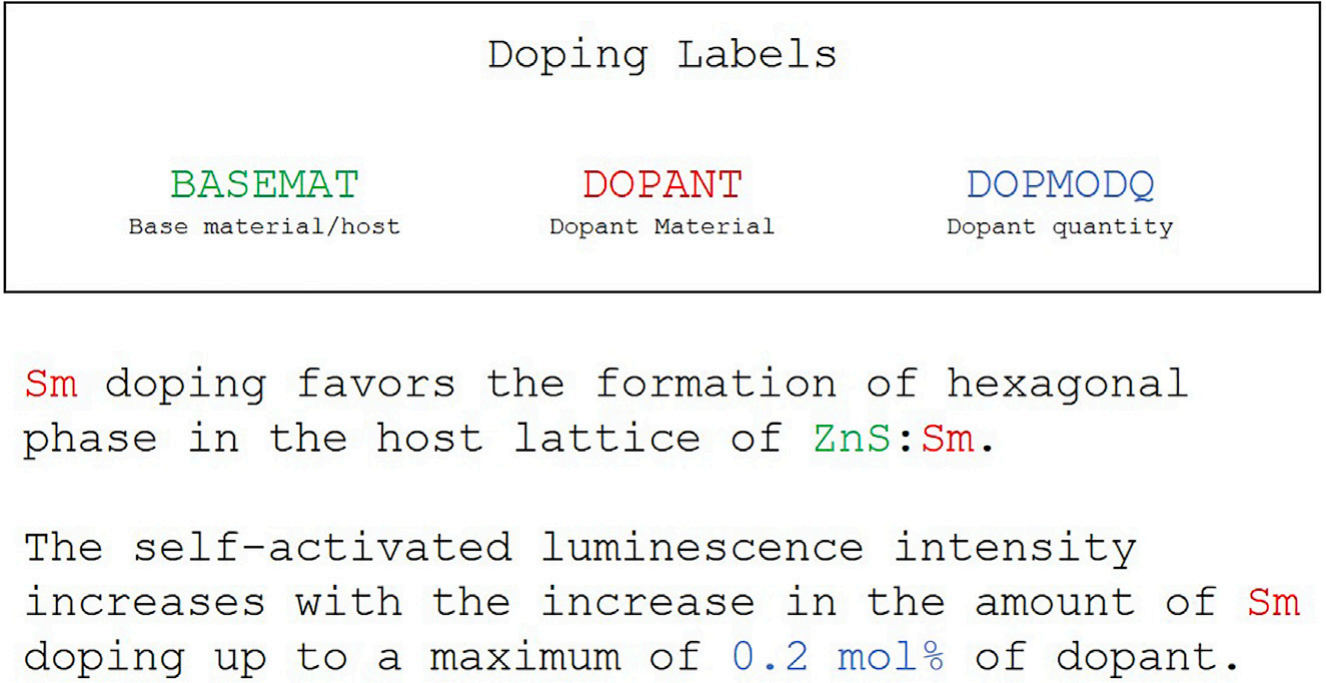
Doping annotation example An example of the doping annotation scheme condensed from an example abstract in the doping dataset. Note that sentence-level annotation was conducted for doping annotations.

A condensed example is shown in Figure 2.^71^ As opposed to the solid-state and gold nanoparticle dataset, tokens were annotated one sentence at a time (one sample = one sentence). Sentences were annotated only when they contain specific and direct information about the doping of solid-state materials, e.g., “X was doped with Y,” “X:Y,” or “Y doping.” Sentences describing byproducts or targeted properties (e.g., magnetization) without direct reference to a dopant or a host material (e.g., “The layered TiO2 phase did not incorporate the dopant specie and had an anatase structure with measured lattice parameters of a=3.61Å, c=9.45Å.”) were not annotated.

#### Gold nanoparticle dataset

Gold nanoparticles (AuNPs) are used widely in biomedicine (e.g., *in vitro* diagnostics), semiconductor technology, and cosmetics.^72^, ^73^, ^74^, ^75^, ^76^ Despite the strong reliance of AuNP properties on size and shape,^77^^,^^78^ only recently have synthesis methods been able to control AuNP morphology, particularly anisotropic nanorods. This dataset aims to capture AuNP morphologies and descriptions from relevant sections of the full text of AuNP synthesis literature. A single annotator annotated a set of 85 characterization paragraphs from 73 articles on AuNP synthesis.

**Figure 3:**
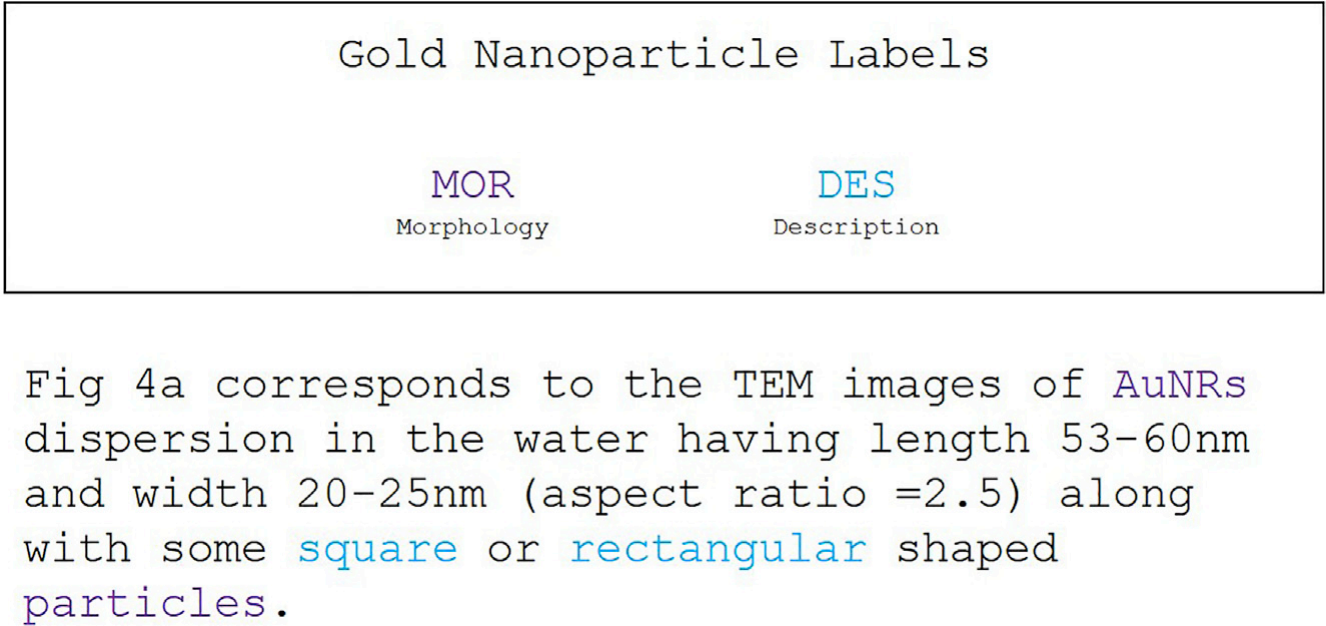
Gold nanoparticle annotation example An example of the gold nanoparticle annotation scheme condensed from an example paragraph in the gold nanoparticle dataset.

A condensed example is shown in Figure 3.^79^ The entities for this model include general shape-based morphological information for the synthesized AuNPs, including noun-based morphological entities (MOR) and adjective-based, descriptive entities (DESs). Entities like “particle” or “AuNP” were annotated as MOR entities, so at least some target could be identified with which to attribute size information in the future since many nanoparticle articles only refer to the particles as the less descriptive “nanoparticle” or “NP.” Note that other aspects such as the dimensions of particles were not included due to very low levels of support for such labels in the original data. This is similar to past work on information extraction from nanomaterial synthesis literature.^57^ Furthermore, limiting the number of labels will tend to provide better performance, particularly for smaller datasets.

### Methods

Four different models are trained and evaluated on each dataset, including a BiLSTM and three variations of networks using the bidirectional encoder representations from transformers (more specifically, BERT) structure. The three BERT networks considered include BERT_BASE_ (uncased), SciBERT (uncased),^21^ and a pre-trained model introduced with this work, MatBERT (uncased). Each model, when given an abstract for a materials science publication in the form of a sequence of tokens, learns to classify each token into pre-defined categories. The token categories correspond to combinations of token position and entity type, i.e., B−MAT, for the beginning token of a material entity. In this way, the NER models described here can be understood as sequence-to-sequence models (Seq2Seq) that transform a sequence of words into a sequence of labels. Unless otherwise specified, for each experiment, 80% of the data was used for training, 10% for validation, and 10% for testing. Sixteen different seeds (integer powers of two from 0 to 15) were used to determine the order of the training data as well as the model weight initialization.

#### Tokenizers

The Materials Tokenizer was used with the BiLSTM model.^28^ First, the tokenization step is carried out using ChemDataExtractor with additional pre-processing to split tokens that are either composed of a number and a unit or an element and a valence state.^80^ Processing the tokens then consists of filtering numbers to become < nUm > since they are often not tokenized correctly with ChemDataExtractor, normalizing simple chemical formulas so the order of the elements is standardized, lowercasing tokens with only the first letter capitalized that are not elements or chemical formulas, and removing accents.

BERT models, however, use the WordPiece subword tokenization algorithm, which is very similar to byte-pair encoding (BPE).^81^^,^^82^ BPE relies on a pre-tokenizer that splits the training data into words. After determining the unique words in the training data and their frequencies, BPE constructs a base vocabulary consisting of all symbols that occur in the words and is trained to learn merging rules, so two symbols from the base vocabulary can be combined to form a new symbol until the vocabulary has grown to the desired size. The learned merging rules can then be applied to new words as long as they are composed of symbols from the base vocabulary. In contrast to BPE, WordPiece learns symbol pairs that maximize the likelihood of the training data rather than the most frequent symbol pairs.

#### Tagging schemes

This work uses the IOBES tagging scheme.^83^ With this scheme, any token that does not correspond to an entity (or part of an entity) is labeled with *O*, denoting an “outside” classification. Single-token entities will be labeled S−X where the S− prefix denotes a “single” token entity, and the *X* is the entity type. For multi-token entities, the prefix B− is used to denote the “beginning” token, E− for the “end” token, and I− for the tokens “inside” the span of the beginning and end tokens. The IOBES tagging scheme has been shown to provide higher F-scores than other similar tagging schemes while retaining the ability to identify consecutive entities.^84^

#### Conditional random field

For all of the models considered, a conditional random field (CRF) is utilized for decoding sequences in addition to calculating the training and validation loss, taking the classification layer output logits as inputs.^85^, ^86^, ^87^, ^88^ As opposed to a classification layer that outputs logits to predict labels without the consideration of neighboring labels, a CRF layer is capable of taking context from these neighboring labels into account when making predictions. Invalid transitions as defined by the tagging scheme (such as I−X being followed by B−X) are initialized to incur large loss penalties.

#### BiLSTM model

The BiLSTM network is an example of a gated recurrent neural network in which the connections between the nodes in the LSTM layers compose a directed graph along a temporal sequence, in this case, a sequence of words. This allows the network to track arbitrarily long-term dependencies in the input sequence, demonstrating temporal dynamic behavior. The bidirectional implementation allows for the LSTM layers to consider both the forward and backward directions of the sequence. Multi-head attention is also used to allow the network to attend to different parts of the sequence differently, i.e., responding to longer-term versus shorter-term dependencies.^11^ These dependency-sensitive representations of the tokens in the sequence can then be used for the downstream classification task via a classification layer. In this work, the word embeddings are initialized using pre-trained Mat2Vec embeddings with a vocabulary size of 529,688.^89^ During training, additional word features are learned using character-level convolutions. These features are then concatenated with the pre-trained Mat2Vec embeddings before being fed into the LSTM layers. The character-level convolutions can aid in improving embeddings for infrequent or even out-of-vocabulary words and have been shown to be useful on relatively small benchmark datasets.^90^

Table 1 summarizes the parameters used to construct the BiLSTM model. The only change in comparison to the BiLSTM model used in past work is the use of convolutional layers instead of BiLSTM layers for the character fields.^28^ For training the BiLSTM model with CRF output and loss, the pre-trained Mat2Vec embeddings were held constant by convention. The RangerLARS optimizer (also known as Over9000),^91^ a combination of a rectified adaptive moment estimation (RAdam)^92^ and Lookahead^93^ to produce the Ranger optimizer^94^ alongside least-angle regression (LARS),^95^ was used for all experiments. A learning rate schedule called “flat and anneal” was utilized, which consists of a constant learning rate for 72% of the training epochs followed by cosine annealing to decay the learning rate to 0.^91^ An initial learning rate of $4\times 10^{-2}$ was used alongside gradient clipping with a maximum norm of 1.0 to prevent exploding gradients. The training was conducted for 64 epochs, and the embeddings were held frozen throughout training.

**Table 1:** BiLSTM parameters: A table of parameters for the BiLSTM model

Word Embedding		Character Embedding		LSTM		Multi-head Attention	
dimension	200	dimension	38	layers	2	heads	16
dropout	0.5	dropout	0.5	hidden dimension	64	dropout	0.25
				dropout	0.1		

#### BERT models

The three BERT models we investigate share the same BERT_BASE_ network structure as well as the same tokenizer algorithm with a maximum vocabulary size of 30,552 tokens. Input sequences are limited to a maximum of 512 tokens. Refer to the original BERT paper for details on its architecture.^9^

Table 2 summarizes the parameters used to construct the BERT_BASE_ model. The three BERT models considered in this work differ only in pre-training, which is largely determined by the corpora on which they are trained. Before training the actual BERT model parameters can take place, the WordPiece tokenizer must be trained on the corpora in order to establish the vocabulary of the model. After the tokenizer is trained, the corresponding BERT model is pre-trained on the same corpora. This consists of two tasks: masked language modeling (MLM) and next sentence prediction (NSP).^9^ The MLM task requires that the BERT model predicts missing words in input sequences where 15% of the words are masked. The NSP task requires that given two sequences, the BERT model predicts the likelihood that one follows the other. It has been shown that pre-training on different corpora can lead to different performances.^21^ This is of particular interest in technical fields where commonly used words and phrases may not be well-represented or even carry the same meaning in other contexts.

**Table 2:** BERT_BASE_ parameters: A table of parameters for the BERT_BASE_ model

Hidden Layers	12	Embeddings	
attention heads	12	hidden dimension	768
dropout	0.1	intermediate dimension	3,072
activation function	GELU	positions	512
layer normalization	$\epsilon =1\times 10^{-2}$	token types	2

The original BERT model was trained on the BooksCorpus (800 million tokens) and English Wikipedia (2.5 billion tokens).^9^ By contrast, SciBERT was trained on 1.14 million scientific papers from Semantic Scholar (3.1 billion tokens) across a variety of fields.^21^ SciBERT was shown to outperform BERT on scientific tasks as a result.

Building on this, we present MatBERT as a BERT model trained using scientific papers specifically from the field of materials science. For training MatBERT, we randomly sampled two million papers, or around 61 million paragraphs, from a corpus mostly consisting of peer-reviewed materials science journal articles.^2^ To optimize MatBERT models for materials science terminologies, two WordPiece tokenizers (cased and uncased) were trained using these paragraphs with no additional pre-processing. Following BERT practices, the vocabulary sizes for the tokenizers are both 30,522. After tokenization, paragraphs with fewer than 20 or more than 510 tokens were removed, leaving a pre-training corpus consisting of around 50 million paragraphs (8.8 billion tokens). The two variants were trained using only the MLM task. An AdamW optimizer was used with a weight decay of 0.01 and the learning rate of $5\cdot 10^{-5}$ decayed linearly to zero during five training epochs. A batch size of 192 paragraphs per gradient update step was used. The convergence of the MLM loss versus training steps can be found in the supplemental information. Each model was trained on eight NVIDIA V100 GPUs and took about 1 month to complete. The pre-training code and pre-trained MatBERT model weights are publicly available.^96^^,^^97^ In this work, the uncased version is used for all BERT variants.

For training of the BERT models (MatBERT, SciBERT, and BERT) with CRF output and loss, the pre-trained model parameters were fine-tuned. The model structures as well as the BERT pre-trained parameters were provided by the “transformers” library.^98^ The SciBERT pre-trained parameters compatible with this library were acquired using the SciBERT AllenAI repository.^21^ All experiments were performed using the PyTorch library.^99^ The LAMB optimizer was used for all experiments.^100^ Different initial learning rates for the BERT embeddings, BERT encoders, and the classification layers (the linear and CRF layers) were employed to reach optimum results. They were respectively chosen as $1\cdot 10^{-4}$, $2\cdot 10^{-3}$, and $1\cdot 10^{-2}$. For the first epoch, only the classification layers are trained, after which the BERT layers are fine-tuned alongside the classification layers for four epochs. For the learning rate schedule, all learning rates are subjected to exponential decay to 10% of the initial value at the final epoch, starting at the end of the second epoch. Gradient clipping with a maximum norm of 1.0 was employed to prevent exploding gradients. For the BERT models (MatBERT, SciBERT, and BERT), the WordPiece tokenizer will often split up words into multiple subtokens. For label predictions, only the embedding of the first subtoken of each word is used for classification. This is consistent with conventional usage.^9^ The code used for training the BERT models on the NER tasks is publicly available.^101^

## Results

In this section, model performances on the aforementioned datasets are reported along with model performance as a function of dataset size. An input sample consists of an entire paragraph from the dataset. The model classification performances are judged according to their achieved precision, recall, and F1-scores using the “micro” averaging scheme to accurately reflect the class imbalances in the datasets. In all experiments, the set of parameters at the end of an epoch that results in the best validation F1-score are evaluated on the test set. In all experiments, training was carried out for 64 epochs for the BiLSTM model and five epochs for the BERT, SciBERT, and MatBERT models. We reiterate that the only difference between the BERT models considered here is the choice of pre-training corpus.

**Figure 4:**
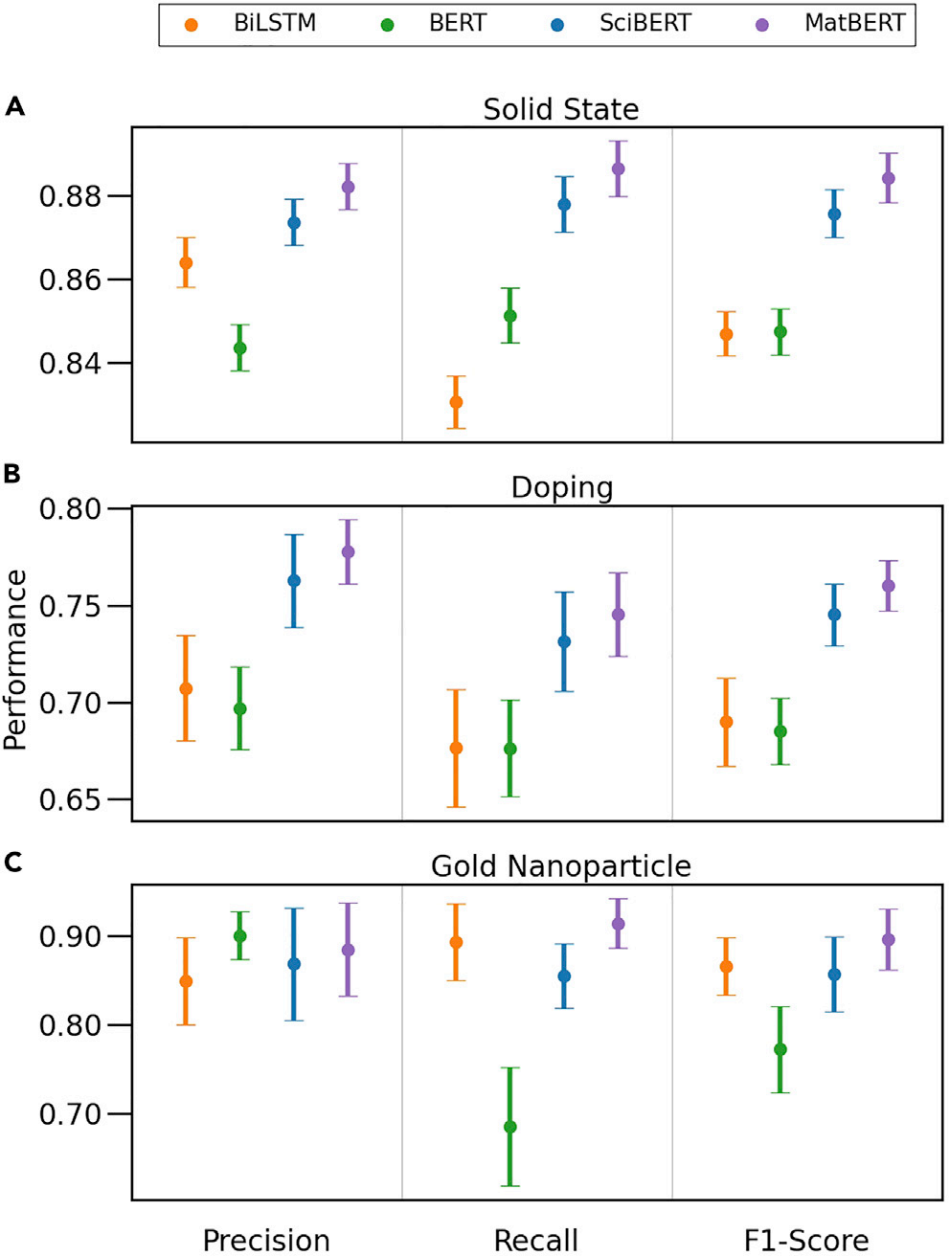
NER model precisions, recalls, and F1-scores Scatterplot summaries of the precisions, recalls, and F1-scores achieved by BiLSTM, BERT, SciBERT, and MatBERT model predictions with respect to the true labels on the solid-state dataset (A), doping dataset (B), and gold nanoparticle dataset (C).

In Figure 4, the performances of the models on the considered datasets are shown. Each point on the scatterplot depicts the 95% CI (assuming a normal distribution) across 16 seeds for the chosen metric, model, and dataset. The precision is the ratio of correctly predicted entities to all predicted entities, and the recall is the ratio of correctly predicted entities to all true entities. The F1-score is the harmonic mean of the precision and recall.

In Figure 4A, it is shown that the MatBERT and SciBERT models perform better than the BERT and BiLSTM models (within statistical error as shown by the CIs) on the solid-state set as determined by the F1-score. For precision, recall, and F1-score, the MatBERT model performs slightly better than the SciBERT model. Interestingly, although the BERT and BiLSTM models achieve very similar F1-scores, there is actually a trade-off between the precision and recall with the models, as the BiLSTM model achieves higher precision, whereas the BERT model achieves higher recall. This means that the BiLSTM model is less susceptible to predicting false positives, while the BERT model is less susceptible to predicting false negatives. The precision and recall are much closer in value for the BERT model than for the BiLSTM model.

Furthermore, in Figure 4B, the same metrics for the doping dataset are shown. Once again, the MatBERT and SciBERT models perform better than the BERT and BiLSTM models. Additionally, the MatBERT model once again demonstrates better performance than the SciBERT model for precision, recall, and F1-score. Compared to the BERT model, the BiLSTM model achieves slightly higher precision (0.71±0.03 versus 0.70±0.02). The respective performances are nearly identical for the recall (0.68±0.03) and F1-score (0.69±0.02). However, the CIs are slightly higher with the BiLSTM model.

Finally, in Figure 4C, the same metrics are once again shown for the gold nanoparticle dataset. The MatBERT model again achieves a higher F1-score than the other models, but for this dataset, the BiLSTM model and the SciBERT model achieve a similar F1-score with the BERT model trailing behind. For the recall, it can be seen that the BERT model performs significantly worse than the other models, with the MatBERT model achieving the best performance followed by the BiLSTM model and then the SciBERT model in turn. For the precision, all of the models perform similarly, with the BERT model actually achieving the best performance, followed by the MatBERT model and then the SciBERT model with the BiLSTM model trailing.

**Figure 5:**
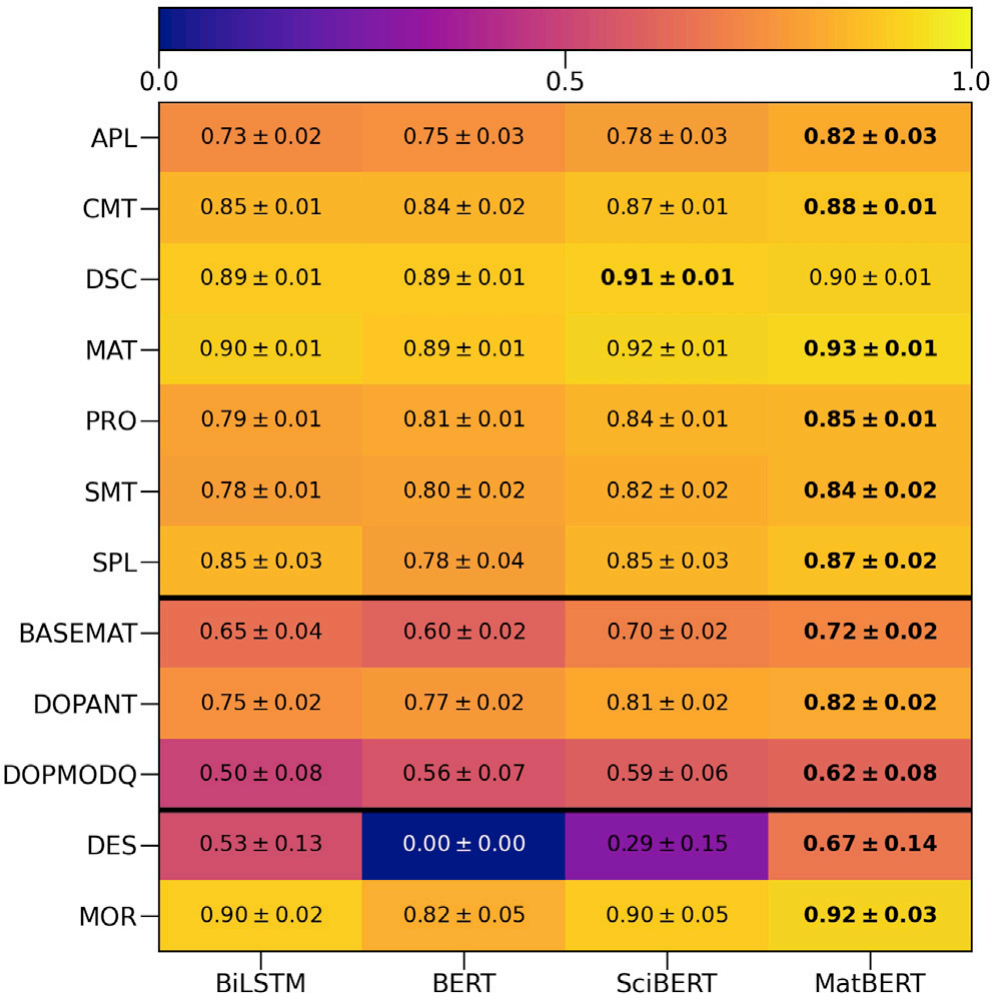
NER model entity score heatmap A heatmap of entity-wise average F1-scores with the best score for each entity in bold.

Figure 5 shows a heatmap of the entity-wise average F1-scores attained for each model across the datasets. The highest score for each entity is in bold. MatBERT claims the best performance for all entities except for one, DSC, where it only slightly lags behind SciBERT. SciBERT then claims the second-best performance for the rest of the entities aside from DES, which the BiLSTM instead claims. Between the BiLSTM and the original BERT, the BiLSTM generally performs better across the entities, only performing much worse compared to BERT for DOPMODQ, slightly trailing behind BERT for the APL, PRO, SMT, and DOPANT entities and performing much better for the solid-state SPL, doping BASEMAT, DES, and MOR entities. Of particular interest is the very poor score of zero obtained by BERT on the DES entity, which was caused by the failure to predict any entities. Since SciBERT also scored poorly on the DES entity (0.29), with the BiLSTM (0.53) and MatBERT (0.67) models significantly outperforming BERT and SciBERT, this would suggest that the domain-specific pre-training is important to DES entity recognition performance.

Generally, the models tend to consistently perform better or worse on the same entities. All of the models tended to perform the poorest on the doping BASEMAT, DOPMODQ, and DES entities and the best on the DSC and MAT entities. There are some exceptions, however, with BERT performing relatively poorly on the SPL and MOR entities despite very good performances from the other models. The model performances on the DES entity vary far more than on the other entities, with very large performance gaps between the models.

**Figure 6:**
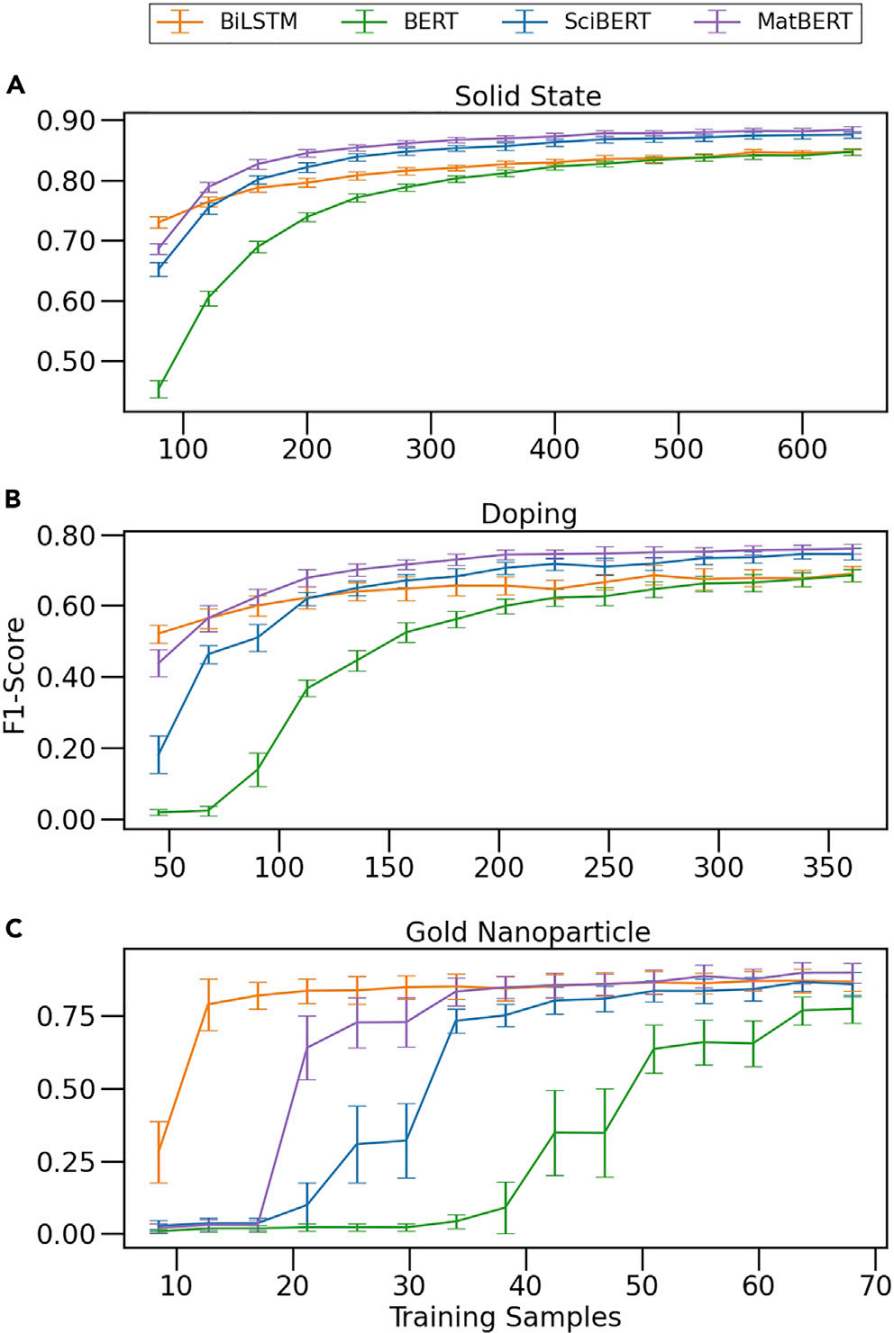
NER model learning curves Learning curves for fine-tuning NER models on the solid-state dataset (A), doping dataset (B), and gold nanoparticle dataset (C). The micro-averaged F1-score on the test set (which is always the same 10% of the total data) is depicted. The smallest training set size was chosen as 10% of the total data and is incremented by 5% up to 80%.

To study the effect of the number of training examples on model performance, we plot learning curves for each model on each dataset in Figure 6. Curating and annotating even modestly sized datasets can entail considerable effort from domain experts in physics, chemistry, and materials science due to the highly technical nature of many publications in those fields. This is in contrast to canonical NER tasks such as CoNLL-2003^102^ (a NER set used in the original BERT publication^9^) that aim to identify less technical entities such as organizations, people, or places. Thus, models that can perform well on small training datasets will be of interest to domain experts looking to create structured technical datasets from text using NER.

In Figure 6, we observe MatBERT and SciBERT exhibiting large performance improvements over BERT at low numbers of training samples, in particular with fewer than 200 samples for the solid-state dataset and with fewer than 50 samples with the gold nanoparticle dataset. The BiLSTM model exhibits the best performance as the training set size approaches zero, but asymptotically approaches a lower limit than the SciBERT and MatBERT models as the number of training points increases. On the solid-state dataset, the larger number of annotated examples allows for BERT to close the gap in F1-score, so the CIs are overlapping at 400 samples and are indistinguishable at 600 samples. As opposed to the SciBERT and MatBERT models, however, BERT does not exceed the BiLSTM performance at any of the training sample intervals for any task. This is not to imply that BERT is approaching the same limit as the BiLSTM; rather, we expect that as the number of training samples is further increased, the general BERT model will exceed or reach the BiLSTM due to its much more complex architecture as seen with the solid-state dataset (though this is less clear for the two smaller datasets). Determining whether adding more NER training data for any one task will outweigh the effects of domain-specific pre-training—that is, whether the general BERT model will overlap SciBERT or MatBERT—requires further investigation with larger numbers of annotated technical text samples. Generally, we observe that more specific pre-training results in increased performance (by substantial margins, e.g., ∼0.05 micro F1-score improvement of MatBERT over general BERT at 320 solid-state training samples) for BERT-derived models at every training set size, particularly at small training set sizes.

**Figure 7:**
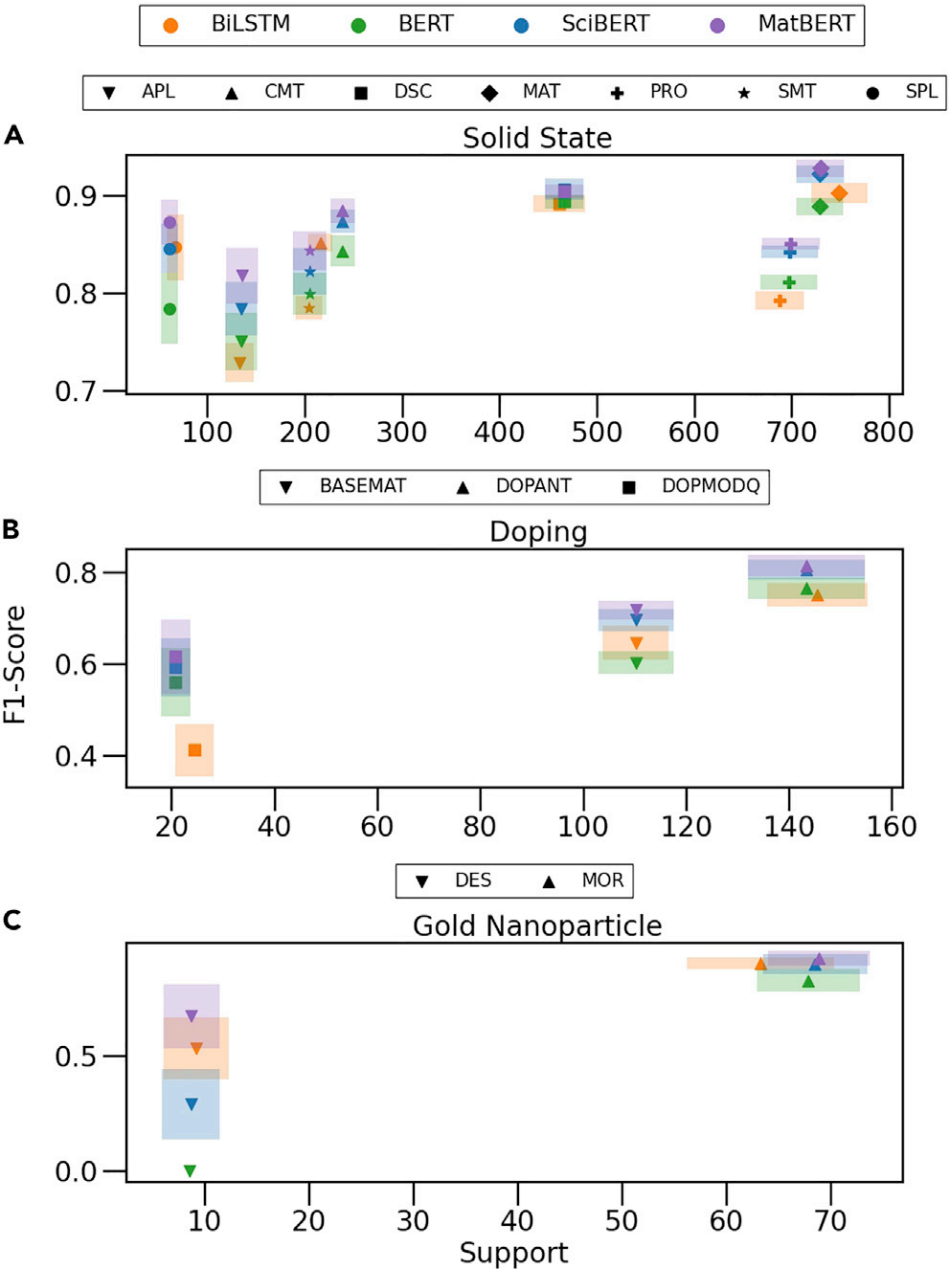
NER model entity scores as a function of support Entity score stratified by label count (support) for each of the datasets. Support varies from model to model due to tokenizer differences that result in different truncations of the input, possibly cutting off some entities. The BiLSTM model imposes no token restriction, while the BERT models are restricted to 512 tokens, with the rest being truncated. Furthermore, different BERT tokenizers can result in a different token count, changing the truncation from model to model.

Another contributing factor to the difference in performance is class support (the number of labels in the testing dataset for a given class). Figure 7 illustrates the disparity among entities’ F1-score by class support for each of the three datasets. As expected, classes with higher support generally have higher F1-scores, and classes with low support stratify according to the level of pre-training. We would intuitively expect MatBERT to perform much better on rarely mentioned entities than BERT given its higher exposure to materials-related text during pre-training. This can be readily seen with the DES entity and DOPMODQ entity, in which model performances likely suffer from very low support (respectively ∼10 and ∼20). For the DES entity, which has the lowest support, the models pre-trained on materials-related text perform significantly better than those trained on general scientific text or just general text. However, the large degree of stratification among BERT models for entities with higher support is of note. Particularly for the PRO entity (e.g., “Voight-Reuss-Hill average bulk moduli”) with a relatively large level of support (∼700 samples), MatBERT and SciBERT both make a substantial ∼0.03 and 0.04 F1-score improvement over BERT. This improvement may imply that highly specialized entities, such as materials science properties that do not appear frequently in general corpora but appear frequently in domain-specific corpora, benefit the most from more specialized pre-training even when there are relatively many samples for fine-tuning. For entities that are more commonly mentioned in general text corpora, such as MOR (e.g., “particles,” “rods,” “spheres”), DOPMODQ (e.g., “3%”), and DSC (e.g., “crystalline,” “amorphous,” “powder”), the level of pre-training appears less important at every level of support.

## Discussion

Whether domain-specific pre-training is needed for large transformer models remains an open question in the field of NLP. Although large models trained on massive general-purpose corpora are complex enough to allow for fine-tuning for various downstream tasks (question/answer, NSP, NER) as opposed to expensive from-scratch retraining, our results show evidence that domain-specific pre-training can measurably improve F1-score performance in the domain of materials science. The overall best performance of MatBERT across the three materials science datasets corroborates a growing body of evidence that domain-specific pre-training is not only a trivial improvement over generally pre-trained models but is indeed worth the effort of retraining large models like BERT. For instance, BioBERT^18^ demonstrated as much as 2.8% F1-score improvement over BERT in the biomedical domain; similarly, both CaseHOLD^19^ (legal corpora) and FinBERT^20^ (financial corpora) yield improvements over base BERT in their respective domains’ downstream tasks. The word distribution shift from a general-purpose corpus to an exclusively technical corpus is large enough to encourage full retraining of large transformer models.

**Figure 8:**
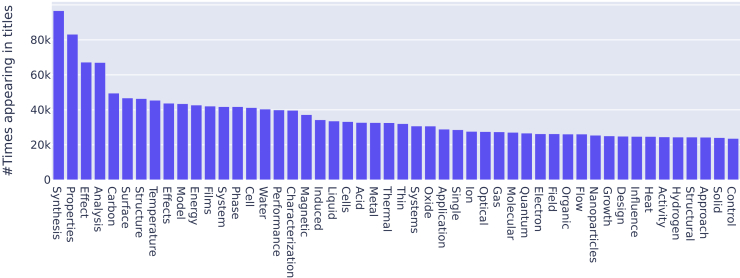
MatBERT keywords Most frequent keywords appearing in the titles of the pre-training corpus of the MatBERT model.

Our results now introduce the question: How specialized should a pre-training corpus be so that it is both highly performant within a domain of knowledge and general enough to address a variety of NER problems within that domain? Although MatBERT improves on BiLSTM, SciBERT, and BERT for all but the smallest training set sizes, the MatBERT model we introduce is limited by the distribution of pre-training data. As detailed in methods, pre-training data were taken from a general material science corpus.^2^ However, as shown by the most frequent title keywords in Figure 8, this corpus is designed to be biased toward trending materials science topics describing experimental syntheses. For example, paragraphs from full texts tend to favor popular compounds (such as oxides, energy materials, and magnetic materials) or synthesis techniques (such as conventional solid-state or hydrothermal synthesis). The MatBERT pre-training corpus, therefore, puts less weight on computational papers containing density functional theory results, theoretical but yet-to-be-synthesized stoichiometries, and unusual but important phase labels. Thus, MatBERT may be improved by expanding the pre-training corpus beyond the set compiled in Kononova et al.^2^ The goal in selecting a pre-training corpus should be to strike a balance between the specificity needed to capture particular facets of materials science and transferability between disparate fields within materials science. Exploring other methods to sample the materials science literature for the purposes of model training is one possible avenue for future work.

### Conclusions

As seen in the presented results and ensuing discussion, the MatBERT model achieves the best overall performance out of the considered models. The 1%∼4% F1-score improvement over SciBERT demonstrates that domain-specific pre-training provides a measurable advantage for NER in materials science. Furthermore, SciBERT improving upon BERT by 3%∼9% F1-score reinforces the importance of scientific pre-training in general for materials science text. Interestingly, it was even found that a comparatively simple BiLSTM model enhanced with embeddings pre-trained on materials science text provides better overall performance than the original BERT model. This suggests that pre-training on a domain-specific corpus can be more impactful on performance than employing modern large transformer-based models. Learning curves additionally show that in the low data limit, the BiLSTM outperforms the BERT models, albeit still with poor overall performance due to the lack of data. For larger datasets, though, MatBERT provides a definitive improvement in NER predictions that can be expected to accelerate the construction of structured materials science datasets.

## Experimental procedures

### Resource availability

#### Lead contact

Requests for additional information should be directed to the lead contact, Nicholas Walker (walkernr@lbl.gov).

#### Materials availability

This study did not generate physical materials.

#### Data and code availability

The pre-trained MatBERT model as well as the trained MatBERT NER models are publicly at https://figshare.com/articles/software/MatBERT-NER_models/15087276.^97^ The code used to pre-train MatBERT is publicly available at https://github.com/lbnlp/MatBERT.^96^ The code used to train MatBERT NER is publicly available at https://github.com/CederGroupHub/MatBERT_NER.^101^ The DOIs of the articles used for the new datasets alongside the associated extracted entities are publicly available at NER Datasets: https://figshare.com/articles/dataset/NER_Datasets_DOIs_and_Entities_Doping_and_AuNP_/16864357.^103^

## Supplemental information

Document S1. Supplemental experimental procedures and Figures S1–S5

Document S2. Article plus supplemental information

## References

[1] Kononova O., He T., Huo H., Trewartha A., Olivetti E.A., Ceder G. (2021). Opportunities and challenges of text mining in materials research. iScience.

[2] Kononova O., Huo H., He T., Rong Z., Botari T., Sun W., Tshitoyan V., Ceder G. (2019). Text-mined dataset of inorganic materials synthesis recipes. Sci. Data.

[3] Olivetti E.A., Cole J.M., Kim E., Kononova O., Ceder G., Han T.Y.-J., Hiszpanski A.M. (2020). Data-driven materials research enabled by natural language processing and information extraction. Appl. Phys. Rev..

[4] Krallinger M., Rabal O., Leitner F., Vazquez M., Salgado D., Lu Z., Leaman R., Lu Y., Ji D., Lowe D.M. (2015). The chemdner corpus of chemicals and drugs and its annotation principles. J. Cheminf..

[5] Gurulingappa H., Mudi A., Toldo L., Hofmann-Apitius M., Bhate J. (2013). Challenges in mining the literature for chemical information. RSC Adv..

[6] Li J., Han P., Ren X., Hu J., Chen L., Shang S. (2021). Sequence labeling with meta-learning. IEEE Trans. Knowl. Data Eng..

[7] Li J., Chiu B., Feng S., Wang H. (2020). Few-shot named entity recognition via meta-learning. IEEE Trans. Knowl. Data Eng..

[8] Li J., Shang S., Chen L. (2021). Domain generalization for named entity boundary detection via metalearning. IEEE Trans. Neural Networks Learn. Syst..

[9] Devlin J., Chang M.-W., Lee K., Toutanova K. (2019). Bert: pre-training of deep bidirectional transformers for language understanding. Preprint at arXiv.

[10] Wang A., Singh A., Michael J., Hill F., Levy O., Bowman S.R. (2019). GLUE: a multi-task benchmark and analysis platform for natural language understanding. Preprint at arXiv.

[11] Vaswani A., Shazeer N., Parmar N., Uszkoreit J., Jones L., Gomez A.N., Kaiser L., Polosukhin I. (2017). Attention Is All You Need. Preprint at arXiv.

[12] Howard J., Ruder S. (2018). Proceedings of the 56th Annual Meeting of the Association for Computational Linguistics (Volume 1: Long Papers).

[13] Peters M.E., Neumann M., Iyyer M., Gardner M., Clark C., Lee K., Zettlemoyer L. (2018). Proceedings of the 2018 Conference of the North American Chapter of the Association for Computational Linguistics: Human Language Technologies, Volume 1 (Long Papers).

[14] McCann B., Bradbury J., Xiong C., Socher R. (2018). Learned in translation: contextualized word vectors. Preprint at arXiv.

[15] Conneau A., Kiela D., Schwenk H., Barrault L., Bordes A. (2017). Proceedings of the 2017 Conference on Empirical Methods in Natural Language Processing.

[16] Zhang K., Bowman S. (2018). Proceedings of the 2018 EMNLP Workshop BlackboxNLP: Analyzing and Interpreting Neural Networks for NLP.

[17] Brown T.B., Mann B., Ryder N., Subbiah M., Kaplan J., Dhariwal P., Neelakantan A., Shyam P., Sastry G., Askell A. (2020). Language models are few-shot learners. Preprint at arXiv.

[18] Lee J., Yoon W., Kim S., Kim D., Kim S., So C.H., Kang J. (2019). Biobert: a pre-trained biomedical language representation model for biomedical text mining. Bioinformatics.

[19] Zheng L., Guha N., Anderson B.R., Henderson P., Ho D.E. (2021). When does pretraining help? assessing self-supervised learning for law and the casehold dataset. Preprint at arXiv.

[20] Araci D. (2019). Finbert: financial sentiment analysis with pre-trained language models. Preprint at arXiv.

[21] Beltagy I., Lo K., Cohan A. (2019). Proceedings of the 2019 Conference on Empirical Methods in Natural Language Processing and the 9th International Joint Conference on Natural Language Processing (EMNLP-IJCNLP).

[22] Li J., Sun A., Han J., Li C. (2020). A survey on deep learning for named entity recognition. Preprint at arXiv.

[23] Eltyeb S., Salim N. (2014). Chemical named entities recognition: a review on approaches and applications. J. Cheminf..

[24] Corbett P., Boyle J. (2018). Chemlistem: chemical named entity recognition using recurrent neural networks. J. Cheminf..

[25] Liang Z., Chen J., Xu Z., Chen Y., Hao T. (2019). A pattern-based method for medical entity recognition from Chinese diagnostic imaging text. Front. Artif. Intelligence.

[26] Sniegula A., Poniszewska-Maranda A., Chomatek L. (2019). Study of named entity recognition methods in biomedical field. Proced. Comp. Sci..

[27] Kanakarajan K.r., Kundumani B., Sankarasubbu M. (2021). Proceedings of the 20th Workshop on Biomedical Language Processing.

[28] Weston L., Tshitoyan V., Dagdelen J., Kononova O., Trewartha A., Persson K., Ceder G., Jain A. (2019). Named entity recognition and normalization applied to large-scale information extraction from the materials science literature. J. Chem. Inf. Model..

[29] He T., Sun W., Huo H., Kononova O., Rong Z., Tshitoyan V., Botari T., Ceder G. (2020). Similarity of precursors in solid-state synthesis as text-mined from scientific literature. Chem. Mater..

[30] Hatakeyama-Sato K., Oyaizu K. (2020). Integrating multiple materials science projects in a single neural network. Commun. Mater..

[31] Dieb T., Yoshioka M., Hara S., Newton M. (2015). Framework for automatic information extraction from research papers on nanocrystal devices. Beilstein J. Nanotechnol..

[32] Gaultois M., Sparks T., Borg C., Seshadri R., Bonificio W., Clarke D. (2013). Data-driven review of thermoelectric materials: performance and resource considerations. Chem. Mater..

[33] Pang N., Qian L., Lyu W., Yang J.-D. (2019). Transfer learning for scientific data chain extraction in small chemical corpus with bert-crf model. Preprint at arXiv.

[34] Corbett P., Copestake A. (2008). Cascaded classifiers for confidence-based chemical named entity recognition. BMC Bioinf..

[35] Krallinger M., Rabal O., Lourenço A., Oyarzabal J., Valencia A. (2017). Information retrieval and text mining technologies for chemistry. Chem. Rev..

[36] Rocktäschel T., Weidlich M., Leser U. (2012). Chemspot: a hybrid system for chemical named entity recognition. Bioinformatics.

[37] Leaman R., Wei C.-H., Lu Z. (2015). tmchem: a high performance approach for chemical named entity recognition and normalization. J. Cheminf..

[38] Korvigo I., Holmatov M., Zaikovskii A., Skoblov M. (2018). Putting hands to rest: efficient deep cnn-rnn architecture for chemical named entity recognition with no hand-crafted rules. J. Cheminf..

[39] García-Remesal M., García-Ruiz A., Pérez-Rey D., De La Iglesia D., Maojo V. (2013). Using nanoinformatics methods for automatically identifying relevant nanotoxicology entities from the literature. Biomed. Res. Int..

[40] Kononova O., He T., Huo H., Trewartha A., Olivetti E.A., Ceder G. (2021). Opportunities and challenges of text mining in materials research. iScience.

[41] Fischer C.C., Tibbetts K.J., Morgan D., Ceder G. (2006). Predicting crystal structure by merging data mining with quantum mechanics. Nat. Mater..

[42] Young S.R., Maksov A., Ziatdinov M., Cao Y., Burch M., Balachandran J., Li L., Somnath S., Patton R.M., Kalinin S.V. (2018). Data mining for better material synthesis: the case of pulsed laser deposition of complex oxides. J. Appl. Phys..

[43] Alperin B., Kuzmin A., Ilina L., Gusev V., Salomatina N., Parmon V. (2016). Terminology spectrum analysis of natural-language chemical documents: term-like phrases retrieval routine. J. Cheminf..

[44] Court C., Cole J.M. (2018). Auto-generated materials database of curie and néel temperatures via semi-supervised relationship extraction. Sci. Data.

[45] Court C., Cole J. (2020). Magnetic and superconducting phase diagrams and transition temperatures predicted using text mining and machine learning. Npj Comput. Mater..

[46] Jessop D.M., Adams S.E., Willighagen E.L., Hawizy L., Murray-Rust P. (2011). Oscar4: a flexible architecture for chemical text-mining. J. Cheminf..

[47] Hawizy L., Jessop D.M., Adams N., Murray-Rust P. (2011). Chemicaltagger: a tool for semantic text-mining in chemistry. J. Cheminf..

[48] Kolářik C., Klinger R., Friedrich C.M., Hofmann-Apitius M., Fluck J. (2008). Workshop on Building and evaluating resources for biomedical text mining.

[49] Mysore S., Jensen Z., Kim E., Huang K., Chang H.-S., Strubell E., Flanigan J., McCallum A., Olivetti E. (2019). The materials science procedural text corpus: annotating materials synthesis procedures with shallow semantic structures, LAW 2019 - 13th Linguistic Annotation Workshop. Proc. Workshop.

[50] Kuniyoshi F., Makino K., Ozawa J., Miwa M. (2020). Annotating and extracting synthesis process of all-solid-state batteries from scientific literature. Preprint at arXiv.

[51] Jensen Z., Kim E., Kwon S., Gani T., Roman-Leshkov Y., Moliner M., Corma A., Olivetti E. (2019). A machine learning approach to zeolite synthesis enabled by automatic literature data extraction. ACS Cent. Sci..

[52] Kim E., Huang K., Saunders A., McCallum A., Ceder G., Olivetti E. (2017). Materials synthesis insights from scientific literature via text extraction and machine learning. Chem. Mater..

[53] Kim E., Jensen Z., van Grootel A., Huang K., Staib M., Mysore S., Chang H.S., Strubell E., McCallum A., Jegelka S. (2020). Inorganic materials synthesis planning with literature-trained neural networks. J. Chem. Inf. Model..

[54] Mysore S., Kim E., Strubell E., Liu A., Chang H.-S., Kompella S., Huang K., McCallum A., Olivetti E. (2017). Automatically extracting action graphs from materials science synthesis procedures. Preprint at arXiv.

[55] Vaucher A., Zipoli F., Geluykens J., Nair V., Schwaller P., Laino T. (2020). Automated extraction of chemical synthesis actions from experimental procedures. Nat. Commun..

[56] Tehseen I., Tahir G., Shakeel K., Ali M., Iliadis L., Maglogiannis I., Plagianakos V. (2018). Artificial Intelligence Applications and Innovations.

[57] Hiszpanski A., Gallagher B., Chellappan K., Li P., Liu S., Kim H., Kailkhura B., Han J., Buttler D., Han T.-J. (2020). Nanomaterials synthesis insights from machine learning of scientific articles by extracting, structuring, and visualizing knowledge. J. Chem. Inf. Model..

[58] Kim J.-D., Ohta T., Tateisi Y., Tsujii J. (2003). Genia corpus – a semantically annotated corpus for bio-textmining. Bioinformatics.

[59] Milosevic N., Gregson C., Hernandez R., Nenadic G. (2019). A framework for information extraction from tables in biomedical literature. IJDAR.

[60] Huo H., Rong Z., Kononova O., Sun W., Botari T., He T., Tshitoyan V., Ceder G. (2019). Semi-supervised machine-learning classification of materials synthesis procedures. Npj Comput. Mater..

[61] Rasmy L., Xiang Y., Xie Z., Tao C., Zhi D. (2020). Med-BERT: pre-trained contextualized embeddings on large-scale structured electronic health records for disease prediction. Preprint at arXiv.

[62] Friedrich A., Adel H., Tomazic F., Hingerl J., Benteau R., Marusczyk A., Lange L. (2020). Proceedings of the 58th Annual Meeting of the Association for Computational Linguistics.

[63] Solid State Abstract Annotations (2019). Solid State Abstract Annotations. https://figshare.com/articles/dataset/Materials_Science_Named_Entity_Recognition_train_development_test_sets/8184428.

[64] Doping and AuNP NER DOIs and Entities (2022). Doping and AuNP NER DOIs and Entities. https://figshare.com/articles/dataset/NER_Datasets_DOIs_and_Entities_Doping_and_AuNP_/16864357.

[65] Elsevier scopus (2022). Elsevier scopus. https://dev.elsevier.com/.

[66] Springer-nature (2022). Springer-nature. https://dev.springernature.com/.

[67] Royal society of chemistry (2022). Royal society of chemistry. https://rsc.org/.

[68] Electrochemical society (2022). Electrochemical society. https://electrochem.org/.

[69] Baek M., Park S., Choi D. (2017). Synthesis of zirconia (zro2) nanowires via chemical vapor deposition. J. Cryst. Growth.

[70] Matscholar (2022). Matscholar. https://matscholar.com/.

[71] Tang T.-P., Yang M.-R., Chen K.-S. (2000). Photoluminescence of zns: Sm phosphor prepared in a reductive atmosphere. Ceramics Int..

[72] Dykman L.A., Khlebtsov N.G. (2011). Gold nanoparticles in biology and medicine: recent advances and prospects. Acta Naturae.

[73] Huang X., El-Sayed M.A. (2010). Gold nanoparticles: optical properties and implementations in cancer diagnosis and photothermal therapy. J. Adv. Res..

[74] Sandeep K., Manoj B., Thomas K.G. (2020). Gold nanoparticle on semiconductor quantum dot: do surface ligands influence fermi level equilibration. J. Chem. Phys..

[75] Lau M., Ziefuss A., Komossa T., Barcikowski S. (2015). Inclusion of supported gold nanoparticles into their semiconductor support. Phys. Chem. Chem. Phys..

[76] Kaul S., Gulati N., Verma D., Mukherjee S., Nagaich U. (2018). Role of nanotechnology in cosmeceuticals: a review of recent advances. J. Pharm..

[77] Dong Y.C., Hajfathalian M., Maidment P.S.N., Hsu J.C., Naha P.C., Si-Mohamed S., Breuilly M., Kim J., Chhour P., Douek P. (2019). Effect of gold nanoparticle size on their properties as contrast agents for computed tomography. Sci. Rep..

[78] Ng S.A., Razak K.A., Aziz A.A., Cheong K.Y. (2014). The effect of size and shape of gold nanoparticles on thin film properties. J. Exp. Nanosci..

[79] Kaur R., Pal B. (2015). Physicochemical and catalytic properties of au nanorods micro-assembled in solvents of varying dipole moment and refractive index. Mater. Res. Bull..

[80] Swain M.C., Cole J.M. (2016). Chemdataextractor: a toolkit for automated extraction of chemical information from the scientific literature. J. Chem. Inf. Model..

[81] Schuster M., Nakajima K. (2012). 2012 IEEE International Conference on Acoustics, Speech and Signal Processing (ICASSP).

[82] Sennrich R., Haddow B., Birch A. (2016). Neural machine translation of rare words with subword units. Preprint at arXiv.

[83] Krishnan V., Ganapathy V. (2005).

[84] Alshammari N., Alanazi S. (2020). The impact of using different annotation schemes on named entity recognition. Egypt. Inform. J..

[85] Lafferty J., McCallum A., Pereira F. (2001). Proceedings of the Eighteenth International Conference on Machine Learning, ICML ’01.

[86] Lample G., Ballesteros M., Subramanian S., Kawakami K., Dyer C. (2016). Neural architectures for named entity recognition. Preprint at arXiv.

[87] Huang W., Cheng X., Wang T., Chu W. (2019). Bert-based multi-head selection for joint entity-relation extraction. Preprint at arXiv.

[88] Souza F., Nogueira R., Lotufo R. (2020). Portuguese named entity recognition using bert-crf. Preprint at arXiv.

[89] Tshitoyan V., Dagdelen J., Weston L., Dunn A., Rong Z., Kononova O., Persson K.A., Ceder G., Jain A. (2019). Unsupervised word embeddings capture latent knowledge from materials science literature. Nature.

[90] Jozefowicz R., Vinyals O., Schuster M., Shazeer N., Wu Y. (2016). Exploring the limits of language modeling. Preprint at arXiv.

[91] Grankin M. (2019). over9000. https://github.com/mgrankin/over9000.

[92] Liu L., Jiang H., He P., Chen W., Liu X., Gao J., Han J. (2020). On the variance of the adaptive learning rate and beyond. Preprint at arXiv.

[93] Zhang M.R., Lucas J., Hinton G., Ba J. (2019). Lookahead optimizer: k steps forward, 1 step back. Preprint at arXiv.

[94] Wright L. (2019). New Deep Learning Optimizer, Ranger: Synergistic Combination of Radam Lookahead for the Best of Both. https://lessw.medium.com/new-deep-learning-optimizer-ranger-synergistic-combination-of-radam-lookahead-for-the-best-of-2dc83f79a48d.

[95] Efron B., Hastie T., Johnstone I., Tibshirani R. (2004). Least angle regression. Ann. Stat..

[96] MatBERT (2021). MatBERT. https://github.com/lbnlp/MatBERT.

[97] MatBERT weights (2022). MatBERT weights. https://figshare.com/articles/software/MatBERT-NER_models/15087276.

[98] Wolf T., Debut L., Sanh V., Chaumond J., Delangue C., Moi A., Cistac P., Rault T., Louf R., Funtowicz M. (2020). Proceedings of the 2020 Conference on Empirical Methods in Natural Language Processing: System Demonstrations.

[99] Paszke A., Gross S., Massa F., Lerer A., Bradbury J., Chanan G., Killeen T., Lin Z., Gimelshein N., Antiga L., Wallach H., Larochelle H., Beygelzimer A., d'Alché-Buc F., Fox E., Garnett R. (2019). http://papers.neurips.cc/paper/9015-pytorch-an-imperative-style-high-performance-deep-learning-library.pdf.

[100] You Y., Li J., Reddi S., Hseu J., Kumar S., Bhojanapalli S., Song X., Demmel J., Keutzer K., Hsieh C.-J. (2020). Large batch optimization for deep learning: training bert in 76 minutes. Preprint at arXiv.

[101] MatBERT (2022). MatBERT NER. https://zenodo.org/badge/latestdoi/315418846.

[102] Tjong Kim Sang E.F., De Meulder F. (2003). http://arxiv.org/abs/cs/0306050.

[103] Doping and AuNP NER DOIs (2022). Doping and AuNP NER DOIs. https://figshare.com/articles/dataset/NER_Datasets_DOIs/16569567.

