# Supplementary material for: Quantifying the advantage of domain-specific pre-training on named entity recognition tasks in materials science
Source: Patterns (N Y). 2022 Apr 8;3(4):100488. doi: 10.1016/j.patter.2022.100488 (PMC9024010; doi:10.1016/j.patter.2022.100488)
Supplement: Document S1. Supplemental experimental procedures and Figures S1–S5 [file mmc1.pdf]

Patterns, Volume *3*

Supplemental information

Quantifying the advantage of domain-specific pre-training on named entity recognition tasks in materials science

Amalie Trewartha, Nicholas Walker, Haoyan Huo, Sanghoon Lee, Kevin Cruse, John Dagdelen, Alexander Dunn, Kristin A. Persson, Gerbrand Ceder, and Anubhav Jain

Supplemental Experimental Procedures

1 CRF

As opposed to a classification layer that outputs logits to predict labels without the consideration of neighboring labels, a CRF layer is capable of taking context from these neighboring labels into account when making predictions. This is done by implementing a linear chain conditional random field that works as a graphical model to introduce dependencies between the predictions in a sequence. The Viterbi algorithm is used to decode the sequences, finding the most likely sequence of classifications while taking into account the dependencies the CRF provides. In order to accelerate early learning, large constant penalties (such as -100) are applied in the CRF transition tensor to discourage the network from violating the rules dictated by the chosen tagging scheme, such as a transition from a beginning ($B-$) tag to and interior tag ($I-$) of a different type.

2 BiLSTM

RNNs with LSTM implementations have enjoyed many successes and reigned for many years in field before the attention concept was proposed, which eventually gave birth to a movement to abandon the RNN approach entirely and instead rely solely on attention-based models. With this approach also comes considerably improved performance, as RNNs are difficult to optimize due to the temporal nature of the backpropagation that cannot be parallelized along the time dimension. By abandoning the RNN approach, attention-based models can be trivially parallelized since attention heads are capable of learning simultaneously. With the focus on self-attention at the core of this architecture, positional embedding additionally needs to be provided in the inputs in order to preserve that sequential information that was being encoded by the LSTM-layers that have now been dropped in favor of attention. The result of this approach culminated in the use of attention-based encoder-decoder architectures called Transformers.[1] Transformer-based networks have empirically shown that attention mechanisms with positional encodings, without recurrent sequential processing are indeed capable of achieving the performance of RNNs with attention.

The BiLSTM network is an example of a gated recurrent neural network (RNN), where the connections between the nodes in the LSTM layers compose

a directed graph along a temporal sequence, in this case a sequence of words. This allows the network to demonstrate temporal dynamic behavior as it keeps track of arbitrary long-term dependencies in the input sequence. The bidirectional implementation allows the LSTM layers to consider both the forward and backward directions of the sequence. In this work, the BiLSTM network also takes advantage of pre-trained word embeddings that provide context-free representations of the words in the vocabulary. It can be intuitively understood that the LSTM layers provide context to the context-free representations of words in a given sequence. Attention is utilized in order to emphasize important parts of the input while de-emphasizing irrelevant or unimportant tokens. This is done in order to alleviate the issues with preserving long-term dependencies in RNN-based models. Intuitively, this can be understood as learning which parts of the sequence are necessary to provide the appropriate context for a particular part of the sequence. Multiple attention heads can be used in similar to that of multiple filters in a convolutional layer: in order to add the capability to respond to different signals in the input. There are two main implementations, additive and dot-product attention, with the latter generally producing better results despite much poorer computational efficiency. The implementation used in PyTorch is dot-product attention. Furthermore, the attention mechanism in consideration is actually self-attention, which refers to the attention being considered for itself as well as other inputs. The end result is a network that, when given an input sequence in the form of temporally-ordered context-free representations of words, learns to classify words in the input sequence by tracking the temporal dependencies between them within the contexts that they appear that produce better predictions.

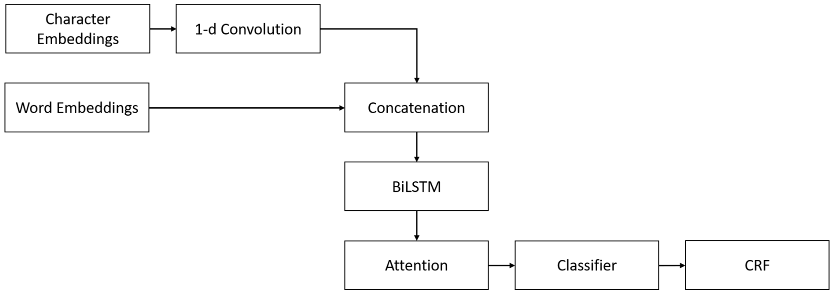

Figure S1: BiLSTM model structure: A diagram of the BiLSTM model structure.

The BiLSTM implementation in this work utilizes the materials tokenizer alongside a vocabulary and associated embeddings provided by Mat2Vec,[2] which is a Word2Vec model specifically trained on materials science texts. The vocabulary size is 529688 with an embedding dimension of 200. Word2Vec models are trained such that the cosine distance between embedding vectors indicate the extent of semantic similarity between the words represented by the vectors. In addition to word embeddings provided via Mat2Vec, trainable char-

acter embeddings are constructed using the character vocabulary of the training set with an embedding dimension of 38. Both word-level and character-level inputs are cased. A dropout ratio of 0.5 is applied to the word and character embeddings. A 1-dimensional convolutional layer is additionally applied to the character embeddings, with 4 filters, a kernel size of $(3,)$, and a kernel stride of $(1,)$. A dropout ratio of 0.25 is applied to the convolution output. The convolution output is then concatenated with the word embeddings to construct word features that utilize features extracted from both Mat2Vec embeddings and convolutions over the words represented as character sequences. Two bidirectional LSTM layer are applied to the word features with a hidden dimension of 64 and a dropout ratio of 0.1. Multihead attention is applied to the bidirectional LSTM output, with an embedding dimension of 128 ($2 \cdot 64$ due to the bidirectional property of the LSTM layer), 16 attention heads, and a dropout ratio of 0.25. This output is then fed into a classification layer with 128 input features and an output feature count equivalent to the size of the label vocabulary. The structure is shown in Fig. S1.

3 BERT

With the rising prominence of Transformer-based models, BERT emerged out of the desire to use a large network of Transformer encoders to encode representations learned from a very large corpus of data. This learning is unsupervised and is meant to produce representations that are very senstitive to contextual information, unlike other context-free unsupervised representation learning approaches such as Word2Vec or GloVe. The representations learned by BERT during pre-training can then be used for learning downstream tasks during training. This approach towards relying heavily on pre-trained representations ideally allows for using a single pre-trained BERT model to perform a variety of downstream tasks without the need to further optimize the BERT parameters while training on said tasks. In practice, however, the pre-trained representations may not always be ideal, though they will often provide a good starting point for further optimization. This approach is very different from older RNN models, in which training is done from scratch aside from perhaps pre-trained context-free word embeddings, though the contextual representations will still need to be trained from scratch even in that case. It has been empirically observed that BERT outperforms RNN-based models on various tasks, providing state-of-the-art performance.[1]

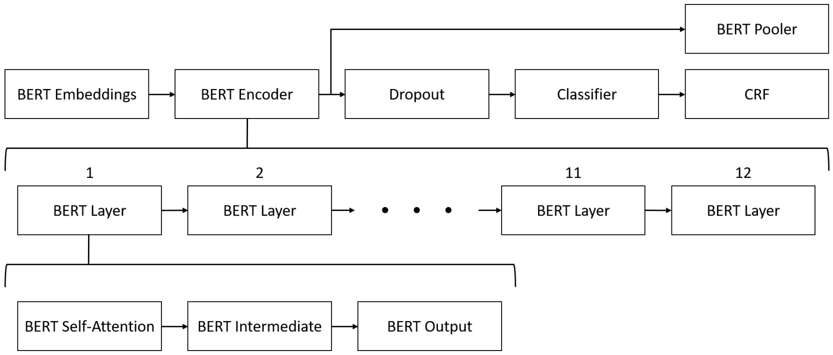

Figure S2: BERT model structure: A diagram of the BERT model structure.

Each pre-trained BERT model consists of both a pre-trained tokenizer as well as pre-trained model weights for a given network structure. The three networks we investigate share the same network structure ($\text{BERT}_{\text{BASE}}$, to be referred to simply as BERT), with 12 hidden layers and 12 attention heads. The embeddings consist of word embeddings with a vocabulary size of 30522 (due to the casing), position embeddings with a maximum of 512 token positions, token type embeddings with a vocabulary size of 2, and layer normalization. Throughout the model, the hidden size is 768, the intermediate size is 3072, the activation function is the Gaussian error linear unit (GELU), the dropout ratio is 0.1, and the layer normalization ϵ is $1 \cdot 10^{-12}$. The 12 hidden layers each consist of three components, an attention layer, an intermediate layer, and an output layer. The attention layer contains both a self-attention layer and a self-output layer. The self-attention layer is composed of three linear layers, respectively corresponding to a query, a key, and a value, as well as a dropout layer. The self-output layer is then composed of dense layer, layer normalization, and a dropout layer. After the attention layer, the intermediate layer consists of a single dense layer. The output layer is identical in structure to the self-output layer with a dense layer, layer normalization, and a dropout layer. After the 12 hidden layers, a pooler layer consisting of a linear layer with hyperbolic tangent (tanh) activation applies a linear transformation over the representation of the first token (CLS) for downstream tasks that are not relevant to the current study. The output of the final hidden layer is then subjected to a dropout ratio of 0.1 and fed into a classification layer with 768 input features (corresponding to the BERT hidden size) and an output feature count equivalent to the size of the label vocabulary. The structure is shown in Fig. S2.

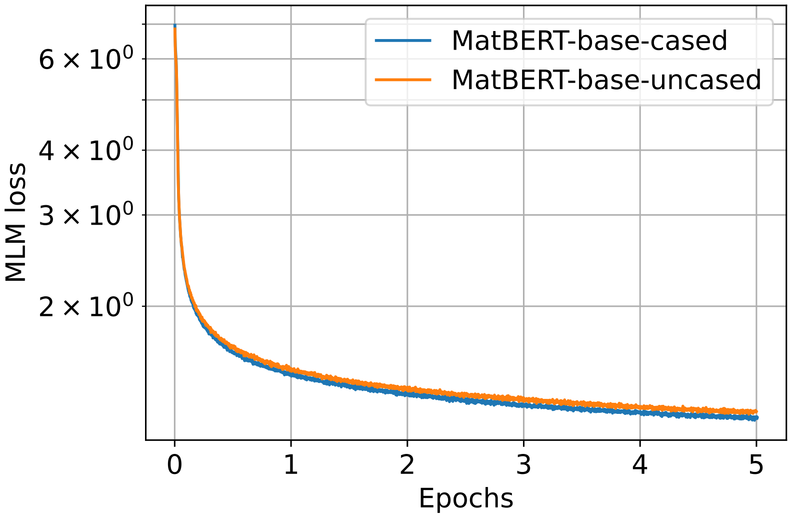

Figure S3: MatBERT training convergence: Masked language modeling (MLM) task loss v.s. training epochs during the pretraining of MatBERT models.

We pretrain MatBERT by optimizing the masked language modeling (MLM) task on 2 million papers, or around 61 million paragraphs, randomly sampled from peer-reviewed materials science journal articles. We used an AdamW optimizer with a weight decay of 0.01 and a learning rate of 5e-5 that decays linearly to zero as the training finishes. The batch size was 192 paragraphs per gradient update step and all models were trained for 5 epochs in total. Each model was trained on 8 NVIDIA V100 GPUs and took about one month to complete. The convergence of MLM loss v.s. training steps is shown in Fig. S3. The training codes and pretrained MatBERT models are publicly available.[3, 4, 5]

4 Uncased and Cased MatBERT Performance

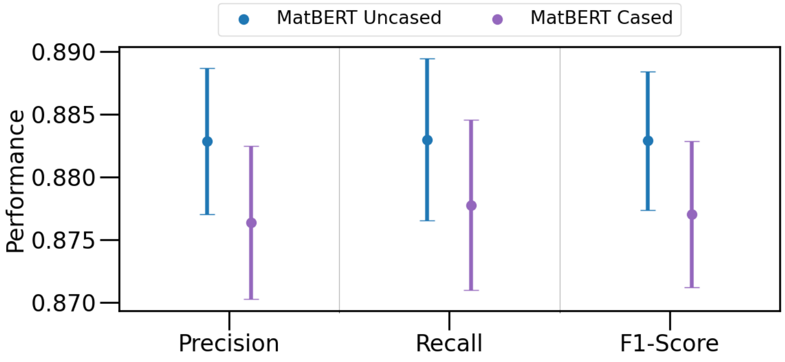

Figure S4: MatBERT uncased vs. cased precision, recall, and F1-score: Scatter plot summaries of the precisions, recalls, and F1-scores achieved by uncased and cased MatBERT model predictions with respect to the true labels on the solid state dataset.

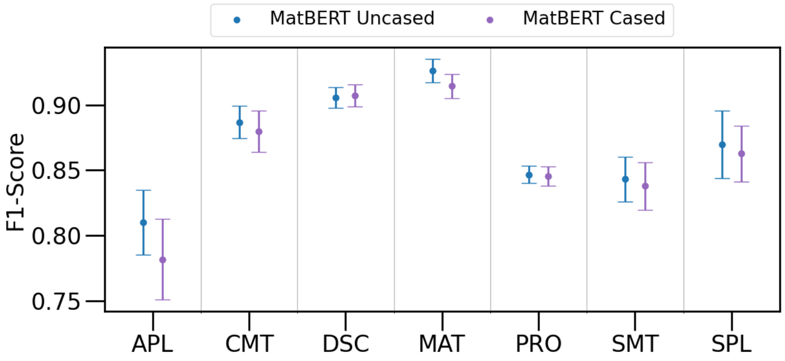

Figure S5: MatBERT uncased vs. cased entity scores: Scatter plot summaries of the entity-wise F1-scores achieved by uncased and cased MatBERT model predictions with respect to the true labels on the solid state dataset.

Performance differences between the cased and uncased MatBERT models are shown in Fig. S4 and Fig. S5. The uncased model performs slightly better and is thus the chosen model for the main manuscript.

References

[1] A. Vaswani, N. Shazeer, N. Parmar, J. Uszkoreit, L. Jones, A. N. Gomez, L Kaiser, and I. Polosukhin. “Attention is All You Need”. In: 2017. arXiv: 1706.03762 [cs.CL].

[2] V. Tshitoyan, J. Dagdelen, L. Weston, A. Dunn, Z. Rong, O. Kononova, K. A Persson, G. Ceder, and A. Jain. “Unsupervised word embeddings capture latent knowledge from materials science literature”. In: *Nature* 571 (2019), pp. 95–98. DOI: 10.1038/s41586-019-1335-8.

[3] *MatBERT*. 2021. URL: https://github.com/lbnlp/MatBERT.

[4] *MatBERT Weights*. 2022. URL: https://figshare.com/articles/software/MatBERT-NER_models/15087276.

[5] *MatBERT NER*. 2022. URL: https://zenodo.org/badge/latestdoi/315418846.
